# Towards Sensor-Based Phenotyping of Physical Barriers of Grapes to Improve Resilience to *Botrytis* Bunch Rot

**DOI:** 10.3389/fpls.2021.808365

**Published:** 2022-02-10

**Authors:** Katja Herzog, Florian Schwander, Hanns-Heinz Kassemeyer, Evi Bieler, Markus Dürrenberger, Oliver Trapp, Reinhard Töpfer

**Affiliations:** ^1^Institute for Grapevine Breeding Geilweilerhof, Julius Kühn-Institut, Siebeldingen, Germany; ^2^Plant Pathology & Diagnostic, State Institute for Viticulture and Enology Freiburg, Freiburg, Germany; ^3^Plant Biomechanics Group & Botanic Garden, Faculty of Biology, University of Freiburg, Freiburg, Germany; ^4^Nano Imaging Lab, Swiss Nano Science Institute, University of Basel, Basel, Switzerland

**Keywords:** high-throughput, berry cuticle, berry texture analysis, QTL, *Botrytis cinerea*, *Vitis vinifera* ssp. *vinifera*, grapevine, phenomics

## Abstract

*Botrytis* bunch rot is one of the economically most important fungal diseases in viticulture (aside from powdery mildew and downy mildew). So far, no active defense mechanisms and resistance loci against the necrotrophic pathogen are known. Since long, breeders are mostly selecting phenotypically for loose grape bunches, which is recently the most evident trait to decrease the infection risk of *Botrytis* bunch rot. This study focused on plant phenomics of multiple traits by applying fast sensor technologies to measure berry impedance (Z_*REL*_), berry texture, and 3D bunch architecture. As references, microscopic determined cuticle thickness (MS_*CT*_) and infestation of grapes with *Botrytis* bunch rot were used. Z_*REL*_ hereby is correlated to grape bunch density OIV204 (*r* = −0.6), cuticle thickness of berries (*r* = 0.61), mean berry diameter (*r* = −0.63), and *Botrytis* bunch rot (*r* = −0.7). However, no correlation between Z_*REL*_ and berry maturity or berry texture was observed. In comparison to the category of traditional varieties (mostly susceptible), elite breeding lines show an impressive increased Z_*REL*_ value (+317) and a 1-μm thicker berry cuticle. Quantitative trait loci (QTLs) on LGs 2, 6, 11, 15, and 16 were identified for Z_*REL*_ and berry texture explaining a phenotypic variance of between 3 and 10.9%. These QTLs providing a starting point for the development of molecular markers. Modeling of Z_*REL*_ and berry texture to predict *Botrytis* bunch rot resilience revealed McFadden *R*^2^ = 0.99. Taken together, this study shows that in addition to loose grape bunch architecture, berry diameter, Z_*REL*_, and berry texture values are probably additional parameters that could be used to identify and select *Botrytis*-resilient wine grape varieties. Furthermore, grapevine breeding will benefit from these reliable methodologies permitting high-throughput screening for additional resilience traits of mechanical and physical barriers to *Botrytis* bunch rot. The findings might also be applicable to table grapes and other fruit crops like tomato or blueberry.

## Introduction

The primary cause of *Botrytis* bunch rot (gray mold) in grapevine (*Vitis vinifera* ssp. *vinifera)* is the necrotrophic cosmopolite fungus *Botrytis cinerea* Pers. ex Fr., the anamorph of *Botryotinia fuckeliana* (de Bary) Whetzel. It is the third economically most important disease in viticulture next to powdery and downy mildew (Dry et al., 2019). Several strategies were developed in the past to control *Botrytis* bunch rot by optimizing viticulture management, plant protection practices, and prediction models (Vail et al., 1998; Valdés-Gómez et al., 2008; Molitor et al., 2011, 2020; González-Domínguez et al., 2015; Fedele et al., 2020). *Botrytis* is a highly adaptable pathogen that is able to rapidly evolve resistance against fungicides (Walker et al., 2017); thus, alternative strategies need to be developed. Management is focused on removing leaves and thinning clusters to reduce moisture content within the bunch zone. Another option is phenotypic or genetic selection of plants resilient to *Botrytis* infestation. In contrast to the mildew fungi, no active defense mechanism by major *R-genes* conferring resistance against *Botrytis* have been found in grapevine or any other crop species (Dry et al., 2019). The lack of active defense against *Botrytis cinerea* is underlined by a study by Rahman et al. (2018) who investigated 81 different grapevine varieties for susceptibility against *Botrytis* leaf spot. Only 14 varieties showed significant degrees of resistance, and the remaining 67 were classified as susceptible; among those, 42 varieties proved to be highly susceptible. For grapevine breeding purposes, early identification and elimination of seedlings susceptible to *Botrytis* infection by marker-assisted selection (MAS) prior to planting into a field would contribute substantially to increase breeding efficiency. Hereby, quantitative trait locus (QTL) mapping is one of the most powerful tools for the development of reliable molecular markers for MAS, e.g., *Rpv*10 (Schwander et al., 2012). Thirteen loci are known to confer resistance to powdery mildew (*Erysiphe necator*), and 28 loci confer resistance to downy mildew (*Plasmopara viticola*) mostly based on *R-gene-*transferred resistance^1^ (Hausmann et al., 2019). QTLs that are linked to *Botrytis* bunch rot resilience are comparably rare (Herzog et al., 2015; Sapkota et al., 2015). Markers for biophysical barriers remain to be developed to early select for the complex trait of *Botrytis* bunch rot resilience in a breeding program.

To reduce the risk of *Botrytis* infections in wine grapes, breeders focus mainly on loose bunch architecture. Berries in loose bunches dry faster after rain events, reducing the risk of berry burst and berry crush that lead to outflow of sugar-rich juice (Percival et al., 1993; Vail et al., 1998; Herzog et al., 2015; Molitor et al., 2018). As a consequence, in loose bunches, the growing conditions for *Botrytis* are less favorable than in compact bunches (Gabler et al., 2003; Töpfer et al., 2011; Rist et al., 2018; Tello and Ibáñez, 2018). Therefore, recent studies started to determine genetic factors associated with loose bunch architecture (Richter et al., 2019, 2020), which opens up the possibility of developing markers for a complex morphological trait in order to early select *Botrytis*-resilient genotypes. However, it can be observed that in some varieties with compact bunches, other traits contribute to reduce *Botrytis* infection risk. It is tempting to speculate that the robustness and permeability of berry skin, which is affected by environmental (biotic and abiotic) factors, also depend also on genetic determinants like thickness of berry skin and thickness of the cuticle. As a necrotrophic fungus and saprophytic colonizer, *Botrytis cinerea* ubiquitously sticks as conidia or sclerotia on all available dead plant tissues (Williamson et al., 2007; Mundy et al., 2012). In spring, growing sclerotia begin to produce conidiophores and conidia as primary sources of inoculum (Williamson et al., 2007). In grapevine as perennial, dead leaves, flowers and mummified fruits contain masses of mycelium ready to produce further conidia (Williamson et al., 2007; Mundy et al., 2012). As soon as micro cracks on berries or other wounds due to berry burst or insect or animal feeding damages emerge, *Botrytis* begins to colonize maturing grape berries (Percival et al., 1993; Commenil et al., 1997; Gabler et al., 2003; Becker and Knoche, 2012; Mundy et al., 2012; Zhang et al., 2020). Deytieux-Belleau et al. (2009) hypothesized that the presence of nutrients and micro-cracks on the surface of maturing berries has a great effect that favors the growth of the pathogen. At this point, the surface of grape berry, i.e., the berry cuticle, comes into play. The cuticle of plants is an extracellular barrier that protects aerial, non-lignified parts of plants from the surrounding environment and from drying, mechanical injuries, and microbial infection (Lara et al., 2019). The cuticle plays an important role in plant pathogen interaction as some cuticle components participate in the activation of plant immune response (Domínguez et al., 2017; Arya et al., 2021). Furthermore, its biomechanical properties are important to prevent fruit cracking under conditions of high humidity, which represents one important starting point for fungal growth (Khanal and Knoche, 2017; Petit et al., 2017; Lara et al., 2019). In contrast to observations that cuticle thickness does not correlate with water permeability (Riederer and Schreiber, 2001), it was shown to be a relevant factor for determining resistance to fungal infections in apple, cranberry (*Vaccinium oxycoccos* L.), stone fruit, and table grapes (reviewed by Lara et al., 2019), and that it influences the susceptibility of grapevines to *Drosophila suzukii* (Weißinger et al., 2021). Furthermore, intra- and epicuticular waxes are extraordinary important, because they fulfill different functions as barriers for water movement (cuticle permeability), resulting in reduced water loss from tissues, and reduced water absorption from the environment, light reflection, and drying of surface (Lara et al., 2019). The ultrastructure of the epicuticular wax is mostly described as a crystalline flake structure and semi-crystalline platelet structure differing in size and distribution of platelets (Percival et al., 1993; Lara et al., 2019; Arand et al., 2021; Yang et al., 2021). These structures assist in repelling water from the surface of the fruit. As concluded by Percival et al. (1993), very large platelet structures on the surface of ‘Cabernet Franc’ are partially responsible for the tolerance of this variety to *Botrytis* bunch rot. In contrast, the highly *Botrytis*-susceptible variety ‘Optima’ showed smaller platelet structures (Percival et al., 1993). Dimopoulos et al. (2020) showed that ‘Merlot’ berries react to water deficiency with increased cuticular wax load and changed cuticular composition, which are both part of the metabolic response of grape berry to drought stress. Additionally, the ultrastructural morphology of cuticular waxes on the surface of the berry changed into larger wax crystals with a more “spindly” and fibrous-like shape (Dimopoulos et al., 2020). Taken together, the cuticle and its hydrophobic epicuticular wax layer display a promising biophysical barrier that can reduce the risk of berry cracking and thus, drastically reduce the risk of spreading *Botrytis* bunch rot infection.

In addition, berry texture traits like berry firmness and berry skin resilience are also described as promising indicators for the susceptibility of grape to *Botrytis* bunch rot of ‘Riesling’ clones (Molitor et al., 2018). In tomato, a relationship between cell wall disassembly of major structural polysaccharides of the cell wall during fruit ripening, and susceptibility to *Botrytis cinerea* was postulated (Cantu et al., 2008). In grape berries, disassembly of the berry cell wall, characterized by decrease in cellulose content and active degradation of xyloglucan and pectin, is responsible for changes in the texture of the berry and softening during ripening (Carreño et al., 2015). In addition, Lara et al. (2019) described a relationship between the cuticle and fruit texture based on a study on tomato, and reports on blueberry (*Vaccinium corymbosum* L.) described cuticle composition and architecture as key factors for ripening-related fruit softening.

In order to dissect these options to understand their contribution to *Botrytis* resilience, efficient phenotyping tools to evaluate the traits are necessary. For high-throughput and precise phenotyping of fruit cuticles and epicuticular waxes, such tools are still in their infancy. Only a handful of methods and protocols focusing on cuticle properties are published. These methods consider individual features like pathogen infection, water loss, and permeability, resulting in phenotyping bottleneck of cuticle-associated traits (Petit et al., 2017). Measurement of the impedance of berries (Z_*REL*_) is an indirect method for the assessment of cuticle thickness and permeability (Herzog et al., 2015). In a proof-of-concept study of Herzog et al. (2015), an easy-to-handle sensor was developed to determine Z_*REL*_ in a high-throughput way. Z_*REL*_ is also proposed as promising indicator for *Botrytis* bunch rot resilience that has to be validated. Furthermore, the mechanical texture of the berry is an important factor for berry firmness. Originally, instrumental texture analysis was performed in food industries to evaluate mechanical and physical characteristics of products (Rolle et al., 2012). Different methods of texture analysis were used for monitoring quality during ripening and postharvest, to investigate grape berry skin and pulp mechanical properties, or to evaluate mechanical berry parameters that increase consumer acceptance of table grapes (Rolle et al., 2012). Penetration (puncture) texture analysis is an established sensor method that is often used to determine table grape firmness or berry skin resilience to insect pests (Letaief et al., 2008; Rolle et al., 2012; Entling et al., 2019). As described by Tonina et al. (2020), the determined texture traits (texture profiles) are related to mechanical features of berry: maximum Force (TA_*FORCE*_) is related to berry skin and external tissue firmness, area (TA_*AREA*_) is related to whole berry consistency/firmness, and gradient (TA_*GRAD*_) is an indicator for berry skin and outer tissue layer elasticity.

In this study, we applied high-throughput sensor techniques to acquire objective and precise phenotypic data in combination with a large-scale genetic study of 364 F1 genotypes for QTL mapping of Z_*REL*_ and berry texture. The study aims at getting a better understanding of the relationship between sensor-based berry traits and *Botrytis* bunch rot resilience as well as the principle usage of such traits in MAS. To meet that aim, the study covers three topics: (i) interaction of Z_*REL*_, grape bunch density and infestation with *Botrytis* bunch rot, as well as the correlation of Z_*REL*_ to other berry traits (e.g., berry texture or cuticle thickness, MS_*CT*_); (ii) evaluation of elite breeding material for Z_*REL*_ and MS_*CT*_; and (iii) multi-trait QTL mapping using the F1 progeny of ‘Dakapo’ × ‘Cabernet Sauvignon’ including the identification of the most relevant berry traits to forecast Botrytis bunch rot resilience.

## Materials and Methods

In order to cover the three stated topics, the study was structured into three tasks:

(i) Investigation of relative berry impedance Z_*REL*_, its correlation to the resilience of grapes to *Botrytis* bunch rot, variety specific differences and correlations to the berry texture.
(ii) Comparison of traditional cultivars, resistant PiWi varieties, and new elite selections regarding Z_*REL*_ and MS_*CT*_.
(iii) QTL mapping of berry skin traits (Z_*REL*_, TA_*FORCE*_, TA_*GRAD*_, TA_*AREA*_).

### Plant Material

Grapes and berries of different categories of plant material were obtained from plants grown in the experimental vineyards at Geilweilerhof, Siebeldingen, Germany (N 49°21.747, E 8°04.678).

- For (i): a set of 26 traditional and PiWi varieties (new fungus-resistant varieties with European Variety Protection), and two resistant accessions from a genetic repository with resistance to powdery and downy mildew;
- For (ii): 80 elite breeding lines, and traditional and PiWi varieties, and
- For (iii): 364 red berry individuals of an F1 population resulting from the cross of ‘Dakapo’ (VIVC14728) and ‘Cabernet Sauvignon’ (VIVC1929), abbreviated as “DxC,” were analyzed.

A PiWi variety is a variety resulting from resistance breeding (regarding resistance to downy and powdery mildew) and harboring resistance traits inherited from American or Asian wild species. Elite breeding lines are an upcoming generation of resistant varieties with higher degrees of resistance. Description details of the used plant material are given in Supplementary Table 1. The description of plant material follows the recommendations of COST Action 17111 Integrape^2^.

All grapevines were grafted on the rootstock Selection Oppenheim 4 [SO4, (VIVC11473) except for the F1 progeny grown on own roots] and trained as a vertical-shoot-positioned trellis system without irrigation as commonly applied in German wine regions. Vines were planted with an inter-row distance of, on average, 2 m and grapevine spacing of m.

### Phenotyping

As berry traits (e.g., firmness of berry skin) and berry health can drastically change because of damages and pathogen infection during ongoing ripening (Rolle et al., 2012), only berries in comparable stage of maturity were considered for phenotyping in this study. Therefore, the BBCH stage of véraison as starting point of the fruit ripening process was scored from each investigated variety and grapevine genotype. Berry sugar content was monitored weekly by Fourier transform infrared spectroscopy (FTIR) or with a manual refractometer.

**FIGURE 1:**
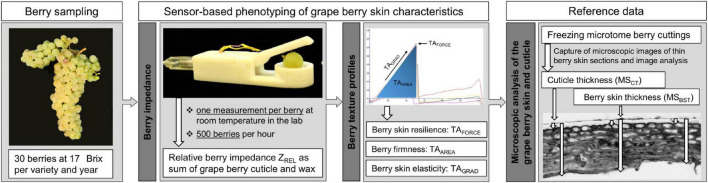
Workflow of phenotyping of different berry skin traits. After berry sampling, berry impedance measurements and texture analysis (TA) were conducted and finally followed by freezing the berries for microscopic analysis.

Phenotypic data recording followed the workflow as depicted in Figure 1.

#### Berry Sampling

From the onset of ripening (véraison), progress in ripening for each genotype was monitored weekly by measurement of sugar content applying a handheld refractometer (VWR^®^ International GmbH, Darmstadt, Germany). A mixture of at least 20 visual unharmed berries per variety (= one replicate) with maturity between 15 and 26°Brix (except three samples with 6, 10, and 12°Brix), were carefully cut at the base of the pedicel in the field or laboratory without touching the surface of the berry. After berry sampling in the morning, samples were transferred to the laboratory where phenotyping was conducted immediately. Sugar content of the investigated berries was measured using a digital refractometer (VWR^®^ International GmbH, Darmstadt, Germany). For QTL analysis and Cryo investigation, three representative grape bunches were sampled right before harvest in the field, and 20 berries were carefully cut in the laboratory. After sampling, the grape maturity of the remaining bunches (sugar content, pH, tartaric acid, and malic acid) was analyzed by FTIR.

#### Berry Impedance

Raw grapevine berry impedance (Z_*BI*_) was determined at 2 and 30 kHz with a fast, low-cost BI sensor following the detailed description of Herzog et al. (2015). Measurement was conducted once per berry at room temperature (±20°C) with a throughput of 500 berries per hour. For one biological replicate per genotype, 15–20 visual intact berries were measured per time point at one randomly chosen point of the lateral berry side, resulting in 20 individual raw Z_*BI*_ values. Relative berry impedance (Z_*REL*_) was calculated for each investigated berry and median for each biological replicate (i.e., 15–20 Z_*REL*_) (Herzog et al., 2015). The number of biological replicates per investigated variety or genotype is given in Supplementary Table 1. After impedance measurement, the berries were directly transferred for the texture analysis procedure in order to prevent water loss or other changes in berry properties.

#### Texture Analysis

An established sensor technology of penetrometer texture analysis was used in order to determine additional phenotypic traits, which are known as indicators for berry skin characteristics. Texture analysis was performed directly after berry impedance measurements at room temperature (±20°C) using the Texture Analyzer TA.XT Express enhanced (Stable Micro System, Goldaming, Surrey, United Kingdom) and a needle probe (P/2N) according to the detailed protocol of Letaief et al. (2008). For each replicate, the berries were measured once at the lateral berry side, resulting in 20 individual texture profiles such as peak positive force (TA_*FORCE*_) as maximum force (time point of berry puncture), area (TA_*AREA*_, Figure 1) as factor describing firmness of the berry, and gradient (TA_*GRAD*_) as berry skin elasticity. For sensor data acquisition and processing, the corresponding software Exponent Lite Express [version 6.1.8.0; Stable Micro Systems, United States] was used.

### 3D Bunch Architecture

Artec^®^ Spider 3D Scanner was used in 2018, 2019, and 2020 within the DxC population according to Rist et al. (2018). For each genotype and year, three representative bunches were harvested and scanned 360° in the laboratory and analyzed automatically with the 3D Bunch Tool as described by Rist et al. (2018). Mean berry diameter (MBD) per bunch was considered.

### Microscopic Investigations

#### Freezing Microtom and Light Microscopic Analysis

In order to determine ground truth data on cuticle (MS_*CT*_) and berry skin thickness (MS_*BST*_), berries were frozen and stored at −20°C after texture analysis. For each replicate, i.e., genotype and year, four berries were used to generate 8-μm thin histological sections of berry skins using Microtom Cryostat Microm HM525 (Thermo Fisher Scientific, Walldorf, Germany) with a trim thickness of 50 μm. For each berry, 24 different berry skin sections were generated, which were screened with a light microscope, Leica DM 4000 B (Leica Microsystems GmbH, Wetzlar, Germany) and 10-fold microscopic magnification. On average, 10 independent sections were used to capture detailed images using the Leica DMC 4500 camera that was mounted on the microscope. Microscopic images of berry skins were analyzed with Leica Application Suite-Modul Interactive Measurement (Leica Microsystems, Hearbrugg, Switzerland). Each of the traits, MS_*CT*_ and MS_*BST*_, was measured three times per image. In total, 12,000 images were analyzed from 300 grapevine replicates in 2018 and 2019, with four berries per replicate (Supplementary Table 1).

#### Cryo Scanning Electron Microscopy

Surface wax structure was analyzed using a scanning electron microscope (Philips XL30 ESEM; Philips) equipped with a cryo preparation unit (Alto 2500; Gatan, United Kingdom). For this purpose, berries of same size were removed from the inside of each grape bunch. Two bunches were used from each genotype. From the berries, a slice of berry skin of approximately 3 mm × 3 mm was excised with a scalpel and mounted with a low-temperature glue on a specimen holder by carefully avoiding touching the waxy surface. Cryofixation was performed with nitrogen slush (<−185°C). The frozen samples were sputtered with 20-nm gold (Au) particles using a high vacuum cryo preparation chamber. The samples were examined with an SE detector operating with acceleration voltage of 5–10 kV at high vacuum and −150°C. In this way, the specimens were cryo-fixed and sputtered, and they were ready for analysis within 90 min after sampling. In total, nine berry samples were analyzed from each variety on one sampling date. The surface structure of each specimen was documented from at least three positions (Arand et al., 2021). Cryo-SEM images were acquired and documented with the DISS5 Software from REM-X GmbH (Bruchsal, Germany).

### Quantitative Trait Locus Mapping

A QTL analysis was performed for all the traits using the mean values for individual years and best linear unbiased predictor (BLUP) values over the years. BLUP is used in linear mixed models to estimate random effects and was calculated with setting year as random effect (cf. 2.7). The DxC map used consists of 739 genotypes evaluated with 270 SSR markers in 19 linkage groups. This resulted in a total map length of 1,500 cM with an average marker distance of 5.5 cM (Schwander et al. unpublished).

MapQTL6.0 (van Ooijen, 2009) was used for the calculation of QTLs. Interval mapping (IM) with 1-cM step size was performed in the first step. A permutation test with 1,000 iterations (*p* < 0.05) was performed for every dataset to determine chromosome-specific and genome wide trait-linked “logarithm of the odds” (LODs) threshold of *p* < 0.05. Genetic regions that exceeded this calculated chromosome-specific LOD threshold are considered as QTLs.

For each QTL, the amount of phenotypic variation and maximum LOD score (LODmax) and its genetic position (LODmax position) are reported. In case of direct LODmax linked marker availability, the marker name is included (Table 2). If no marker was linked to the QTL, the next available marker was stated.

### Phenotyping *Botrytis* Bunch Rot Infestation

Scoring of *Botrytis* bunch rot infection was performed in 2017 and 2021 in the field. Plant material was selected in the experimental vineyards and genetic repository at JKI Geilweilerhof (DEU098) with at least three plants per accession (Supplementary Table 1). Varieties with additional damages like berry shrivel, symptoms of downy or powdery mildew (leaves and grapes), or insect damages were excluded. After sampling for berry impedance measurement at approximately 17°Brix, *Botrytis* bunch rot of grapes was scored at harvest maturity using 5-class classification (class 1: no infection; class 3: low infection; class 5: medium infection; class 7: high infection; class 9: very high infection) as described in detail by Herzog et al. (2015). One scoring value was determined for each replicate at harvest and year. In total, 57 replicates were evaluated, i.e., 25 replicates in 2017 and 32 replicates in 2021.

### Statistics

Statistical data analysis and generation of graphical figures were conducted using open source software R version 3.6.3 (R Core Team, 2019) and RStudio (version 1.2.5019). Pearson correlations were conducted with mean phenotypic values of Z_*REL*_, TA_*FORCE*_, TA_*GRAD*,_ TA_*AREA*_, MS_*CT*_, and MS_*BST*_ per genotype and year. Therefore, the R library (Hmisc) package and rcorr function were used (Harrell, 2021). Tukey HSD was applied to compare the different groups of *Botrytis* bunch rot-susceptible genotypes and traditional varieties, PiWis, and breeding material with regard to Z_*REL*_, grape bunch architecture, and/or MS_*CT*_. For the calculation of the best linear unbiased predictor (BLUP), the package ‘nlme’ was used (Pinheiro, 2021) setting genotype as fixed effect and year as random effect. For *Botrytis* bunch rot prediction, the package ‘nnet’ was used to fit multinominal model via neural networks (Ripley, 2013). Furthermore, the fit of the logistic model was tested using the package ‘DescTool’ and Pseudo *R*^2^ McFadden (*R*^2^*_*McF*_*) was calculated (Signorell, 2021).

## Results

### Z_*REL*_ Is Correlated to *Botrytis* Bunch Rot, Grape Bunch Architecture, and Cuticle Thickness

**FIGURE 2:**
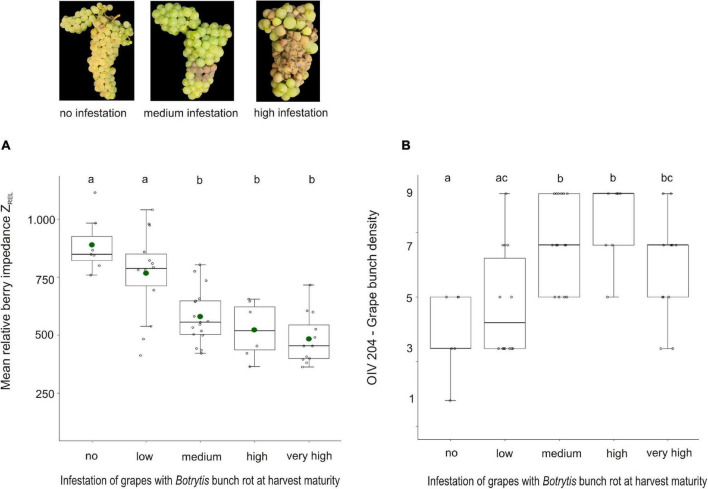
Mean berry impedance Z_*REL*_ **(A)** and grape bunch density factor OIV 204 **(B)** of 28 varieties with, on average, 17°Brix in relation to the infection of grapes with *Botrytis* bunch rot at harvest in seasons 2017 and 2021. Samples were grouped according to infection with *Botrytis* bunch rot that was scored right before harvest. Detailed information about underlying varieties, year of investigation, their stage of maturity at harvest, and *Botrytis* infection is given in Supplementary Table 2. *N* (= replicates) per infection group: “no” = 7, “low” = 14, “medium” = 17, “high” = 7, and “very high” = 12. Mean Z_*REL*_ and *Botrytis* bunch rot infection was determined in one to six replicates per variety (see Supplementary Table 2). Different letters indicate significant differences. Green dots display the mean values. *N* = 57.

In 2017 and 2021, natural grape infection with *Botrytis* bunch rot was scored in the field when the grapes were ready for harvest (Supplementary Table 2). These infestation data were used to clarify the relationship between Z_*REL*_ and grape bunch architecture (OIV 204), and *Botrytis* bunch rot susceptibility (Figure 2). Significant differences of mean Z_*REL*_ were detected between the *Botrytis* bunch rot-resilient (no to low infestation) and susceptible (medium to very high infestation) groups (Figure 2A). Here, the resilient group (= no *Botrytis* bunch rot), for instance, showed a mean Z_*REL*_ that is 406 units higher than that of the highly susceptible group (Supplementary Table 3). With focus on median bunch density OIV 204, the group with no *Botrytis* bunch rot was classified as class 3 (loose grape bunch architecture). In the *Botrytis* bunch rot-susceptible (medium to very high infestation) group, median bunch density was classified as class 7 (compact grape bunch architecture), i.e., bunches in the susceptible group were, on average, denser than those in the resilient group (Figure 2B and Supplementary Table 2).

Pearson correlation of Z_*REL*_ and OIV 204 with *Botrytis* bunch rot revealed *r* = −0.7 for Z_*REL*_ and *r* = −0.41 for grape bunch density OIV 204. Both traits, Z_*REL*_ and OIV 204, are also correlated to each other (*r* = −0.6). Further, Z_*REL*_ and OIV 204 were tested in order to predict the risk for *Botrytis* bunch rot. Within the data set (Supplementary Table 2), Z_*REL*_ could be confirmed as an objective berry trait to predict *Botrytis* bunch rot resilience (*R*^2^*_*McF*_* = 0.28), while the combination of Z_REL_*OIV 204 increased the prediction accuracy to *R*^2^*_*McF*_* = 0.43 (Supplementary Table 4). Considering maturity at harvest into the model (Z_REL_*OIV 204*Brix) revealed *R*^2^*_*McF*_* = 0.66. Based on these results, we concluded that the looser the grape bunch architecture and the higher the berry impedance value Z_*REL*_, the lower the risk for infection with *Botrytis* bunch rot.

**FIGURE 3:**
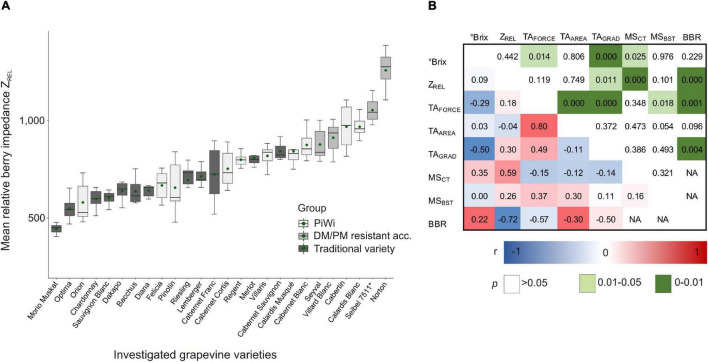
Boxplot of relative berry impedance (Z_*REL*_) of “traditional” (trad) and PiWi varieties (PiWi) as well as downy mildew- and powdery mildew-resistant grapevine accessions **(A)** and Pearson correlation of different berry skin traits and *Botrytis* bunch rot **(B)**. **(A)** *N* = 6 replicates per variety (i.e., 3 years as shown in Supplementary Table 1, two replicates per year, and 15 berries per replicate); **(B)** *N* = 73 replicates (*N* = 41 replicates for MS_*CT*_ and MS_*BST*_; *N* = 32 replicates for *Botrytis* bunch rot infection). PiWi, new resistant grapevine varieties; DM/PM, downy mildew/powdery mildew; acc., accession; °Brix, berry sample sugar content; TA, texture analysis; TA_*FORCE*_, berry skin firmness; TA_*AREA*_, whole berry firmness; TA_*GRAD*_, berry skin elasticity; MS_*CT*_, cuticle thickness; MS_*BST*_, berry skin thickness; BBR, *Botrytis* bunch rot. Details are given in Supplementary Tables 5–7.

In a 3-year study, the phenotypic variation of berry impedance was investigated in the ripening stage of approximately 17°Brix using 26 grapevine varieties (Figure 3A). Furthermore, Z_*REL*_ was correlated to further berry traits, i.e., berry maturity (°Brix), berry texture, and berry skin as well as cuticle thickness (Figure 3B). The correlation between the berry texture traits TA_*FORCE*_, TA_*AREA*_, and TA_*GRAD*_, and *Botrytis* bunch rot infection is also shown in Figure 3B.

The phenotypic expression of berry impedance varies between Z_*REL*_ = 445 (‘Morio Muskat’ as traditional variety) and 1,258 (‘Norton’ as resistant interspecific crossing of *V. aestivalis* and *V. vinifera*), as shown in Figure 3A (phenotypic data are given in Supplementary Table 5). The mean relative Z_*REL*_ of all the investigated PiWi varieties reaches, on average, 794, which is 1.2-fold higher than that of the investigated traditional varieties, with an average Z_*REL*_ of 657. Within the traditional variety compilation, ‘Morio Muskat,’ ‘Optima,’ ‘Chardonny,’ ‘Sauvignon Blanc,’ ‘Dakapo,’ ‘Bacchus,’ and ‘Dian” showed a mean Z_*REL*_ lower than 650, and no values were higher than 842 (‘Cabernet Sauvignon’) (Figure 3A and Supplementary Table 5). In the PiWi group, only ‘Orion,’ one of the first PiWi varieties, showed Z_*REL*_ values lower than 650, while ‘Calardis Blanc’ and ‘Cabertin’ showed the highest berry impedance values of Z_*REL*_ = 969. In this set of varieties (Supplementary Table 7), Z_*REL*_ is correlated to microscopic cuticle thickness *r* = 0.59 (*p* = 0.025) but is not correlated to sugar content or berry texture (Figure 3B). For berry texture, only berry skin elasticity (TA_*GRAD*_) is correlated to sugar content (Brix); concurrently TA_*FORCE*_ and TA_*GRAD*_ are significantly correlated to *Botrytis* bunch rot.

### Elite Breeding Lines Show Robust Cuticular Properties

**FIGURE 4:**
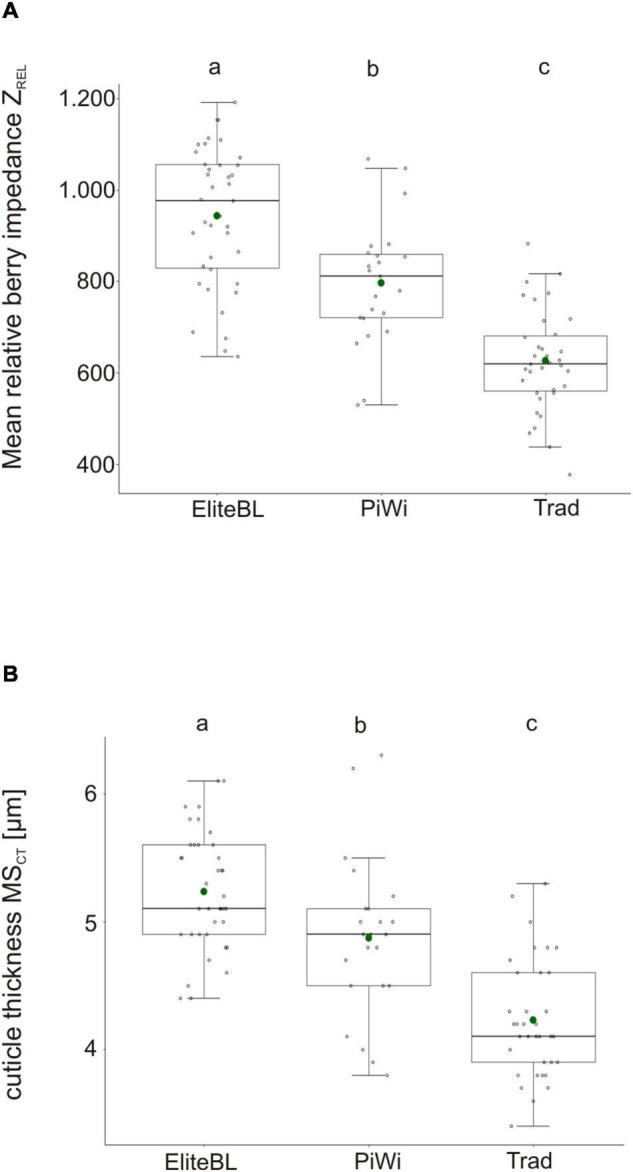
Boxplot of the mean relative impedance Z_*REL*_ **(A)** and mean cuticle thickness MS_*CT*_ **(B)** in elite breeding lines (Elite BL), PiWi (fungus-resistant varieties), and traditional varieties (Trad). Different letters indicate significant differences. *N* = 39 (Elite BL), *N* = 35 (Trad), and *N* = 23 (PiWis). Detailed information about investigated accessions is given in Supplementary Table 8.

In addition to Z_*REL*_, the cuticle thickness (MS_*CT*_) was measured microscopically and was compared between the set of 41 grapevine varieties and 39 elite breeding lines (Figure 4).

Both cuticular traits, berry impedance Z_*REL*_ and cuticle thickness MS_*CT*_ were significant higher in elite breeding lines (group elite BL) in comparison to traditional and PiWi varieties (Figure 4). The selected elite breeding material showed a mean Z_*REL*_ = 948 [Z_*REL*_ = 648 (min) and Z_*REL*_ = 1,190 (max)]. For instance, established traditional varieties showed −317 smaller Z_*REL*_ value and on average −1.0 μm thinner cuticles compared with the investigated elite breeding lines (Supplementary Table 8). The PiWi varieties were grouped between traditional varieties and elite breeding lines. Remarkably, 71% of the breeding lines showed Z_*REL*_ values >850 and 48% even Z_*REL*_ > 1,000.

### Berry Skin Phenomics Reveals New Insights Into Underlying Genotypic Relationships

#### Screening for One Optimal Mapping Population Based on the Ultrastructure of Surface Waxes, Berry Impedance, and Berry Texture

In the first step, parental varieties of two mapping populations (‘Riesling’ × ‘Sauvignon Blanc’ and ‘Dakapo’ × ‘Cabernet Sauvignon’) were phenotyped once in 2019 regarding their berry skin characteristics at harvest maturity (Table 1). ‘Morio Muskat’ (with smallest Z_*REL*_, Figure 3A) and ‘Norton’ (with highest Z_*REL*_, Figure 3A) were used as extremes (Figure 3A). In addition, Seibel 7511, ‘Calardis Blanc’ and ‘Cabernet Blanc’ were selected as *Botrytis* bunch rot-resilient genotypes (Supplementary Table 2) with differences in Z_*REL*_, berry texture, and cuticle thickness.

**TABLE 1:**
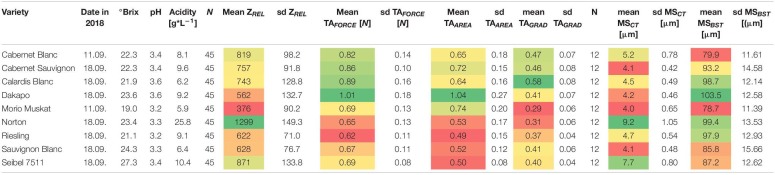
Phenotypic characteristics of berry skin and ultrastructure of epicuticular waxes of selected varieties.

*For comparison, grape must parameters are shown. The color gradient indicate the level of phenotypic values within the dataset from red (low level) to dark green (high level).*

As shown in Table 1, the *Botrytis*-resilient variety ‘Norton’ shows the highest mean values of Z_*REL*_ and MS_*CT*_ (Supplementary Tables 2, 5, 6). In contrast, ‘Morio Muskat’ as a *Botrytis*-susceptible variety shows the lowest values for Z_*REL*_ and MS_*CT*_. Surprisingly, the berry texture properties of both varieties were comparable to berry skin resilience (TA_*FORCE*_) and berry elasticity (TA_*GRAD*_). ‘Calardis Blanc’ and ‘Cabernet Blanc’ showed comparable values for berry texture, while Z_*REL*_ and MS_*CT*_ were a little higher in ‘Cabernet Blanc.’ ‘Cabernet Sauvignon’ and ‘Dakapo’ showed comparable MS_*CT*_ but contrasting Z_*REL*_ and berry texture. ‘Riesling’ and ‘Sauvignon Blanc’ showed comparable values for Z_*REL*_ and berry texture, and cuticle MS_*CT*_ was thicker in ‘Riesling.’ However, to be sure that the F1 progeny of the selected population is segregating regarding Z_*REL*_ and berry texture, parental genotypes should show different phenotypes.

**FIGURE 5:**
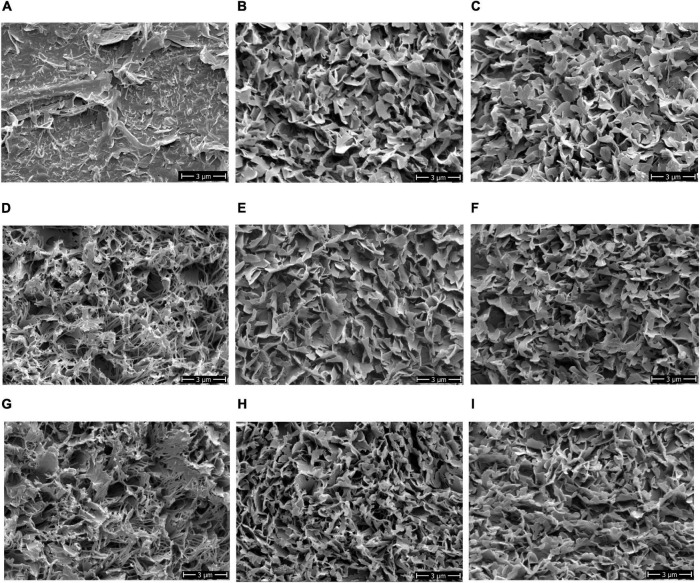
Ultrastructural phenotype of the epicuticular waxes on the berry surfaces of different grapevine varieties. Exemplary images illustrate the structural differences: **(A)** ‘Morio Muskat,’ **(B)** ‘Riesling,’ **(C)** ‘Sauvignon Blanc,’ **(D)** ‘Calardis Blanc,’ **(E)** ‘Dakapo,’ **(F)** ‘Cabernet Sauvignon,’ **(G)** ‘Norton,’ **(H)** Seibel 7511, and **(I)** ‘Cabernet Blanc.’ Cryo-SEM 8,000x. SEM, scanning electron microscope.

In addition to berry impedance, berry texture, and microscopic cuticle thickness, the ultrastructure of the epicuticular waxes on the berry surface was investigated in parallel. Therefore, Cryo-SEM analysis on the grape berry surface of the ripe bunches was conducted to investigate visual differences in the ultrastructure of epicuticular waxes (Figure 5).

Four categories of crystalline wax structures on the berry surfaces of the investigated varieties were identified: (1) fragmented; (2) large and (3) fine platelets, and (4) thin, fibrous-like wax crystals (Figure 5). The berry surface of ‘MorioMuskat’ was characterized by the absence of coherent wax platelets and some kind of wax fragments (Figure 5A). ‘Riesling,’ ‘Sauvignon Blanc,’ ‘Dakapo,’ and ‘Cabernet Sauvignon’ showed large crystalline platelets, whereas Seibel 7511 and ‘Cabernet Blanc’ showed an appearance of more fine structures. ‘Calardis Blanc’ and ‘Norton,’ in contrast, appeared particularly different with very thin, fibrous-like wax crystals. Regarding berry skin properties (Table 1), Z_*REL*_ (*r* = 0.81), TA_*GRAD*_ (*r* = 0.43), and MS_*CT*_ (*r* = 0.65) were correlated to the observed wax category. The phenotypes for berry texture TA_*FORCE*_, TA_*AREA*_, and TA_*GRAD*_ of *Botrytis*-resilient ‘Norton’ is comparable to the berry texture values of the *Botrytis*-susceptible ‘MorioMuskat’ and ‘Sauvignon Blanc’ or Seibel 7511. ‘MorioMuskat’ without typical wax structure had comparable cuticle thickness as ‘Cabernet Sauvignon,’ ‘Sauvignon Blanc’ or ‘Dakapo.’ Even the cuticle thickness of ‘Calardis Blanc’ was comparable to that of ‘Riesling’ but they differed substantially in Z_*REL*_, TA_*FORCE*_, TA_*AREA*_, TA_*GRAD*_ as well as the ultrastructure of epicuticular waxes.

In 2019, the wax crystal structure of ‘Riesling,1 ‘Calardis Blanc’ and ‘Norton’ berries appeared comparable to the structures observed in 2018, whereby the berries showed comparable maturity stage (Supplementary Figure 1).

For QTL mapping of Z_*REL*_ and berry texture, varieties were selected differing in both traits but showing similar wax structure to avoid unexpected effects. Based on their phenotypic variance regarding Z_*REL*_ and berry texture, the F1 mapping population of ‘Dakapo’ × ‘Cabernet Sauvignon’ was used for QTL analysis in this study.

#### QTLs of Z_*REL*_ and Berry Texture Are Affected by Berry Size and Teinturier Phenotype

**FIGURE 6:**
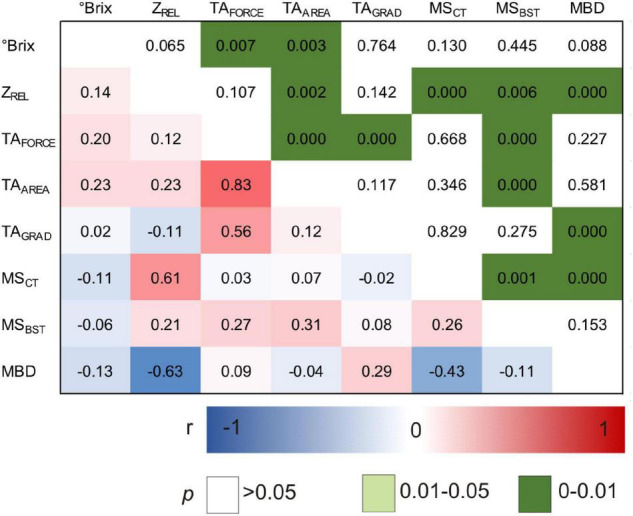
Pearson correlation matrix of investigated phenotypic traits within the subset of ‘Dakapo’ × ‘Cabernet Sauvignon’ mapping population. *N* = 180 genotypes. °Brix, berry sample sugar content; Z_*REL*_, berry impedance; TA, texture analysis; TA_*FORCE*_, berry skin firmness; TA_*AREA*_, whole berry firmness; TA_*GRAD*_, berry skin elasticity; MS_*CT*_, cuticle thickness; MS_*BST*_, berry skin thickness.

The F1 progeny of ‘Dakapo’ × ‘Cabernet Sauvignon’ (D × C) was phenotyped at harvest between 2018 and 2020 for sensor-based berry (skin) traits (Z_*REL*_, TA_*FORCE*_, TA_*AREA*_, and TA_*GRAD*_), and microscopic reference analysis of cuticle thickness (subset of 180 genotypes once in 2018, MS_*CT*_ and MS_*BST*_) as well as mean berry diameter (MBD) (Figure 6). All the genotypes expressed red-colored berries. The data obtained from all the investigated traits were normally distributed. All the traits segregated within the population of D × C (Supplementary Figure 2).

As shown in Figure 6, correlations are identified between Z_*REL*_ and MS_*CT*_ (*r* = 0.61, *p* < 0), mean berry diameter (MBD) (*r* = −0.63, *p* < 0), and a small but significant correlation is observed between Z_*REL*_ and berry firmness TA_*AREA*_ (*r* = 0.23, *p* < 0.002). MS_*CT*_ was correlated to MBD (*r* = −0.43, *p* < 0.000). While MS_*CT*_ did not correlate to any of the texture profiles of TA_*FORCE*_, TA_*AREA*_, and TA_*GRAD*_, MS_*BST*_ did at least to TA_*FORCE*_ and TA_*AREA*_. Sugar content (Brix) showed minor correlation to TA_*FORCE*_ and TA_*AREA*_ but no correlation to Z_*REL*_ or MS_*CT*_ as previously observed in the variety study (Figure 3B).

The mean values per genotype and year as well as the BLUP values of all the investigated years per genotype were used for QTL analysis. Interval mapping (IM) resulted in 18 QTLs for Z_*REL*_, TA_*FORCE*_, TA_*GRAD*_, TA_*AREA*_, and MBD, which were distributed in 13 linkage groups and were stable in 2 or 3 years (Supplementary Table 9). Only QTLs (BLUP values) that additionally exceeded the genome wide LOD threshold are shown in Table 2.

**TABLE 2:** Important quantitative trait loci (QTLs) (MQM method) identified for best linear unbiased predictor (BLUP) values of berry impedance (Z_*REL*_), berry texture of TA_*FORCE*_, TA_*GRAD*_, and TA_*AREA*_, and mean berry diameter (MBD).

Trait* (+ co-variant)	Co-factor	LG	LOD_*max*_ position	LOD value	% expl. variation	SSR marker (Pos. [cM])	Physical position on PN40024 12xV2 (bp)	Conf. Interval (LOD_*max*_^–1^ [cM])	Conf. Interval (LOD_*max*_^–2^ [cM])	Flanked marker downstream (Pos. [cM])	Physical position on PN40024 12xV2 (bp)	Flanked marker upstream (Pos. [cM])	Physical position on PN40024 12xV2 (bp)
Z_*REL*_	GF11-05 + GF15-34 + GF02-55 + VRZAG15 + UDV-085	2	56.0	14.1	10.1	GF02-55 (57.5)	chr02_14110164	53.0–57.5	51.0–57.5	VMC5G7 (51.0)	chr02_8335117	GF02-62 (58.0)	chr02_14304052
		6	29.2	5.5	3.8	UDV-085 (28.2)	chr06_4906382	24.1–33.2	22.1–33.2	GF06-04 (22.1)	chr06_3797817	VMC2G2_127 (33.4)	chr06_5819580
		11	47.0	16.4	12.0	GF11-05 (45.0)	chr11_7856900	44.3–49.0	43.3–49.0	VCHR11A (43.3)	chr11_7418122	GF11-09_311 (49.3)	chr11_10465761
		15	64.4	10.8	7.6	GF15-34 (61.4)	chr15_15913905	51.5–69.4	47.5–69.4	GF15-04 (42.5)	chr15_14298293	VMC4D9.2 (70.3)	chr15_16581058
		17	35.5	14.0	10.1	VRZAG15_162 (35.5)	chr17_6588726	32.4–39.5	30.4–40.5	VVIN73 (28.4)	chr17_5636281	GF17-11 (45.9)	chr17_8820821
TA_*FORCE*_	GF02-62	2	58.0	15.9	18.4	GF02-62 (58.0)	chr02_14304052	57.5–58.0	–	GF02-55 (57.5)	chr02_14110164	VVNTM6_144 (58.3)	chr02_14442196
		15	65.4	4.1	4.2	GF15-34 (61.4)	chr15_15913905	52.4–85.9	42.4–85.9	GF15-04 (42.5)	chr15_14298293	VCHR15A (85.9)	chr15_19977880
TA_*AREA*_	GF02-55	2	56.0	29.3	31.1	GF02-55 (57.5)	chr02_14110164	55.0–57.5	54.0–57.5	VMC5G7 (51.0)	chr02_8335117	GF02-62 (58.0)	chr02_14304052
		15	72.3	5.0	4.3	GF15-05 (70.3)	chr15_17079049	56.5–83.8	47.5–85.9	GF15-04 (42.5)	chr15_14298293	VCHR15A (85.9)	chr15_19977880
TA_*GRAD*_	GF16-52 + GF02-62	2	58.0	7.8	8.0	GF02-62 (58.0)	chr02_14304052	57.5–58.0	–	GF02-55 (57.5)	chr02_14110164	VVNTM6_144 (58.3)	chr02_14442196
		16	44.1	13.1	13.9	GF16-52_112 (43.1)	chr16_17255388	42.2–47.1	42.2–50.1	GF16-12_334 (42.2)	chr16_16472526	GF16-18 (50.7)	chr16_18294446
MBD	VRZAG15 + GF02-55 + GF11-05	2	57.5	12.4	11.2	GF02-55 (57.5)	chr02_14110164	55.0–57.5	54.0–57.5	VMC5G7 (51.0)	chr02_8335117	GF02-62 (58.0)	chr02_14304052
		11	44.3	10.3	9.1	GF11-05 (45.0)	chr11_7856900	43.3–49.0	–	VCHR11A (43.3)	chr11_7418122	GF11-09_311 (49.3)	chr11_10465761
		17	35.4	14.5	13.3	VRZAG15_162 (35.5)	chr17_6588726	31.4–38.5	29.4–40.5	VVIN73 (28.4)	chr17_5636281	GF17-11 (45.9)	chr17_8820821
Z_*REL*_ + MBD + TP	VCHR06A_186 + GF11-05 + GF15-34_273 + SCU06_163	6	39.6	5.8	3.3	VCH06A_186 (38.6)	chr06_7188641	33.4–41.6	–	VMC2G2_127 (33.4)	chr06_5819580	VVIC50 (42.3)	chr06_8450955
		11	47.0	11.4	6.7	GF11-05 (45.0)	chr11_7856900	43.3–49.0	–	VCHR11A (43.3)	chr11_7418122	GF11-09_311 (49.3)	chr11_10465761
		15	56.5	8.5	4.9	GF15-34 (61.4)	chr15_15913905	47.5–68.4	42.5–69.4	GF15-04 (42.5)	chr15_14298293	VMC4D9.2 (70.3)	chr15_16581058
		17	16.5	5.6	3.2	SCU06_163 (16.5)	chr17_3290363	13.2–21.5		VMC3C11.1 (13.2)	chr17_2296673	UDV-072(21.9)	chr17_4390242
TA_*FORCE*_ + MBD + TP	GF02-62	2	58.0	5.6	5.5	GF02-62 (58.0)	chr02_14304052	57.5–58.0	–	GF02-55 (57.5)	chr02_14110164	VVNTM6_144 (58.3)	chr02_14442196
		15	72.3	4.6	4.3	GF15-05 (72.6)	chr15_17079049	57.5–76.6	50.5–85.9	GF15-04 (42.5)	chr15_14298293	VCHR15A (85.9)	chr15_19977880
TA_*AREA*_ + MBD + TP	GF02-62	15	73.5	5.5	4.5	GF15-05 (72.6)	chr15_17079049	58.5–81.8	53.5–85.9	GF15-04 (42.5)	chr15_14298293	VCHR15A (85.9)	chr15_19977880
TA_*GRAD*_ + MBD + TP	GF16-52 + GF02-62	2	58.0	5.9	5.5	GF02-62 (58.0)	chr02_14304052	57.5–58.0	–	GF02-55 (57.5)	chr02_14110164	VVNTM6_144 (58.3)	chr02_14442196
		16	42.2	11.2	10.9	GF16-12_334 (42.2)	chr16_16472526	42.2–47.1	42.2–50.1	GF16-12_334 (42.2)	chr16_16472526	GF16-18 (50.7)	chr16_18294446

*QTL analysis was additionally conducted by setting MBD and TP as co-variants. TP, Teinturier phenotype; TA, texture analysis; TA_FORCE_, berry skin firmness; TA_AREA_, whole berry firmness; TA_GRAD_, berry skin elasticity; LG, linkage group; LOD, logarithm of the odds; SSR, simple sequence repeats; cM, centimorgan; bp, base pairs; conf., confidence.*

MQM resulted in five QTLs for Z_*REL*_ with LOD_*max*_ values of 5.5–16.4, two QTLs for TA_*FORCE*_ with LOD_*max*_ values of 4.1–15.9, two QTLs for TA_*AREA*_ with LOD_*max*_ values of 5–29.3, two QTLs for TA_*GRAD*_ with LOD_*max*_ values of 7.8–13.1, and three QTLs for berry size MBD with LOD_*max*_ between 10.3 and 14.5 (Table 2). As MBD was significantly correlated to Z_*REL*_ and TA_*GRAD*_ (Figure 6) and as overlaying QTLs were detected for both, berry skin traits and MBD, MBD was set as co-variant within MQM mapping. Furthermore, LG 2 is involved in the phenotypic expression of all the investigated traits. As the detected markers GF02-55 (PN40024.V2, located on chromosome 02: 14.11 Mb, Table 2) and GF02-62 (PN40024.V2, located on chromosome 02: 14.30 Mb, Table 2) are co-localized to the berry color locus *Gret1*(PN40024.V2, located on chromosome 02: 14.24 Mb (Pelsy et al., 2015), we included the Teinturier phenotype in our QTL analysis. The Teinturier phenotype is characterized by red berry flesh and red leaves (Röckel et al., 2020). In the investigated DxC progeny, genotypes expressing the Teinturier phenotype showed significant but marginal higher mean Z_*REL*_, TA_*FORCE*_, and TA_*AREA*_ values as well as lower berries and TA_*GRAD*_ (Supplementary Figure 3). Pearson correlation revealed correlations between the Teinturier phenotype of *r* = −0.19 to TA_*GRAD*_, *r* = 0.34 to Z_*REL*_, and up to *r* = 0.55 for TA_*AREA*_ (Supplementary Table 10). Based on the observed correlation of the physical traits to MBD and Teinturier phenotype, both were set as co-variants, resulting in decreased LOD_*max*_ values for all the QTLs on LG 2, Z_*REL*_ on LGs 11, 15, and 17 (LG 2 disappeared), and for TA_*GRAD*_ on LG 16 (Table 2). Notably, only TA_*FORCE*_ and TA_*GRAD*_ mapped at the berry color locus on LG 2 within a confidence interval of 0.3 Mbp. In addition to the locus on LG2, QTLs and LOD_*max*_ positions were often similar for different berry skin traits: Z_*REL*_ and berry texture (TA_*FORCE*_, TA_*AREA*_, and TA_*GRAD*_) mapped both on LG15; Z_*REL*_ and berry size on LG11 and LG17. However, for the development of molecular markers and their application in marker-assisted-selection (MAS) in grapevine breeding, the importance of the specific traits to select for *Botrytis* bunch rot resilience is needed. Therefore, the 2021 data set (Supplementary Table 2) was used to model Z_*REL*_, berry texture profile (TA_*FORCE*_, TA_*AREA*_, and TA_*GRAD*_) and mean berry diameter (MBD) in order to predict *Botrytis* bunch rot. The combination of Z_*REL*_ and MBD (Z_REL_*MBD) revealed a *R*^2^*_*McF*_* = 0.69, while the combination of Z_*REL*_ and berry texture profile (Z_REL_*TA_FORCE_*TA_AREA_*TA_*GRAD*_) increased the explained variance to *R*^2^*_*McF*_* = 0.99. In conclusion, the model indicates that screening or MAS of these traits could be a novel way to select for *Botrytis* bunch rot-resilient genotypes.

## Discussion

### Berry Impedance as an Indicator for Resilience to *Botrytis* Bunch Rot and Its Relationship to Berry Texture Traits

In viticulture, management practices are an important option to reduce the risk of *Botrytis* infection. This includes application of Botryticides or treatments that aim at faster drying of grapes such as mechanical thinning (Schwendel et al., 2021), Gibberellin application (variety-dependent) (Hed and Centinari, 2021) or early defoliation (at flowering) (English et al., 1989; Molitor et al., 2011) to generate looser grape bunch architecture. Late defoliation (around veraison) supports faster drying of the bunch zone. In addition to faster drying, these management treatments enable better fungicide coverage. From a point of view of sustainable production, undoubtedly, treatments that promote faster drying of bunches are superior and, in particular, relevant for a pathogen like *Botrytis*, which is highly adaptable and proved to easily build up resistance against plant protection agents. However, additional labor costs need to be considered as well. Therefore, grapevine breeders look for loose bunch architecture as the most important selection criterion to improve resilience to *Botrytis* bunch rot for faster drying and decreased risk for berry damages. Recently, efforts were made to genetically dissect bunch architecture (Vail et al., 1998; Correa et al., 2014; Rist et al., 2018; Richter et al., 2019; Tello et al., 2020). However, loose bunch architecture does not always guarantee *Botrytis* resilience. Especially, unfavorable weather conditions, i.e., continuous rainfall resulting in high air humidity and constant wetness during berry ripening, support uncontrolled spreading of *Botrytis* bunch rot. Thus, additional factors and their genetic basis that contribute to strengthen the plant for *Botrytis* resilience remain to be identified. In this study, the berry impedance Z_*REL*_ was validated by the investigation of more varieties for over 3 years. By testing an extended set of genotypes and additional seasons, our results confirmed the findings by Herzog et al. (2015) that Z_*REL*_, especially in combination with loose grape bunch architecture and high berry texture values is reliable and suitable for screening of *Botrytis* bunch rot resilience. In the investigated set of varieties, the group of varieties with no *Botrytis* infestation showed high mean Z_*REL*_ (890), whereas the varieties with high or very high *Botrytis* infestation exhibit low mean Z_*REL*_ (500). The 3-year study has demonstrated that the varieties ‘Sauvignon Blanc,’ ‘Chardonnay,’ and ‘Optima’ as well as ‘Morio Muskat,’ ‘Orion,’ ‘Bacchus,’ ‘Dakapo,’ and ‘Diana’ had Z_*REL*_ values below 650. Based on the findings shown in Figure 2, varieties with Z_*REL*_ values lower than 650 and dense grape bunch architecture can be classified as *Botrytis*-susceptible. This confirms reports in which ‘Sauvignon Blanc,’ ‘Chardonnay,’ and ‘Optima’ are described as susceptible varieties (Percival et al., 1993; Molitor et al., 2011, 2018; Schwendel et al., 2021). In contrast, Z_*REL*_ values >900 or >1,000 will increase the probability of *Botrytis* resilience of grape berries independent of its bunch architecture (cf. Supplementary Table 2). Such grapes are likely to remain resilient even under high infection conditions, e.g., with long period of wetness, as observed in Seibel 7511 (dense grape bunch architecture, Z_*REL*_ > 1,000, and no *Botrytis* infection). The accession of ‘Norton,’ which is known as *Botrytis-*resilient (Sapkota et al., 2019) was the cultivar with the highest Z_*REL*_ value (>1,000). However, some varieties like ‘Cabernet Franc,’ ‘Cabernet Cortis’ and ‘Cabertin’ showed stronger fluctuations in Z_*REL*_ values between replicates and years. There was no extraordinary difference in maturity between years or weather conditions before sampling (Supplementary Table 3). This variance might result from the location of the bunch in the canopy, decreased cell vitality, or damages due to insects (Caravia et al., 2015) or non-visible micro cracks.

Especially with focus on reliable sensor-based prediction of *Botrytis* resilience of breeding lines, a better understanding of physical berry impedance in relation to other traits becomes necessary. The correlation study (Figure 3B) at the variety level revealed that Z_*REL*_ is positively correlated to cuticle thickness and independent from the maturity stage (°Brix) of the berries. However, despite observations in tomato and blueberry that the cuticle (cuticle thickness and deposition) is related to fruit texture (Lara et al., 2019), in our study, grape berry texture did not correlate with Z_*REL*_ or cuticle thickness but is also negatively correlated to *Botrytis* bunch rot. Berry skin elasticity (TA_*GRAD*_) is negatively correlated to the maturity stage of the berries, which can be explained because of stronger berry softening during ripening. This observation can be explained by the disassembly of berry flesh cell walls during ripening, which is responsible for berry softening and, thus, changes in the texture of the berry (Carreño et al., 2015). Furthermore, berry skin elasticity is weakly correlated to berry skin resilience TA_*FORCE*_, because in some varieties, especially ‘Dakapo,’ berry flesh is very soft, resulting in a more flexible berry behavior.

### Elite Breeding Material

Traditional varieties are the reference for comparative phenotyping of a new elite breeding material toward *Botrytis* resilience. As berry impedance is an additional means to categorize breeding lines for *Botrytis* resilience, we determined Z_*REL*_ in the elite breeding material of our breeding program. The new elite breeding lines showed a high mean Z_*REL*_ of 948 compared to traditional varieties (with Z_*REL*_ = 621). It is remarkable that 48% of the breeding lines showed Z_*REL*_ values >1,000. These values are comparable to those of the berry impedance of the ‘Norton’ variety (Figure 3A), which is described as *Botrytis*-resilient (Sapkota et al., 2015). ‘Norton’ has never shown any *Botrytis* infection in the field yet (data not shown). Besides berry impedance, the elite breeding lines also showed cuticle thickness MS_*CT*_ = 5.2 μm, which is, on average, 1-μm thicker than that of traditional varieties. Conclusively, today’s elite breeding lines show a more robust berry surface characterized by very high Z_*REL*_ values and thicker cuticle (Supplementary Table 8). Gabler et al. (2003) noticed that the berries of American *Vitis* species with cuticle thickness of 4 to 10 μm show more *Botrytis* resilience as the European *V. vinifera* with cuticle thickness of up to 3.8 μm. Conclusively, the investigated elite breeding lines show an increased probability to be *Botrytis*-resilient. The crosses of investigated lines were conducted in 2011, and seedlings were evaluated between 2014 and 2017. In years 2014 (Herzog et al., 2015) and 2017, high *Botrytis* infection pressure was present because of high air humidity of up to 87% over a long period and precipitation sum between 31 and 90 mm in the ripening months August/September. Nevertheless, the elite breeding lines were selected during the breeding process while they were showing low infection with *Botrytis* bunch rot during such challenging environmental conditions (data not shown). Thus, the breeder indirectly selected for robust berry surface, i.e., high Z_*REL*_.

### Differences in the Ultrastructure of Epicuticular Waxes

The grapevines examined in this study showed differences in the surface properties of the grapevine berries. Assuming that Z_*REL*_ is additionally influenced by epicuticular waxes, we selected nine grapevine varieties (including the resistant genotype Seibel 7511) based on their variance in Z_*REL*_. Four different types of ultrastructures were observed: (1) no wax, large (2) crystalline platelets, (3) fine crystalline platelets, and (4) very thin, fibrous-like wax crystals. For ‘Riesling,’ ‘Calardis Blanc’ and ‘Norton,’ we observed a comparable ultrastructure in 2018 and 2019 (Supplementary Figure 1). Especially, thin, fibrous-like wax structures were previously described as a result of water stress in grapevine (Dimopoulos et al., 2020) but not as characteristic of different varieties. In addition to the environmental effect (Dimopoulos et al., 2020) and time of berry development (Arand et al., 2021), we conclude that the variety might have an impact on wax composition and, thus, wax structure. Heredia-Guerrero et al. (2018) reviewed for plant cuticles in general that both increased temperatures and drought affect cuticles by increase in main cuticle components, cutin and polysaccharides, as well as changes in the chemical composition of waxes, i.e., accumulation of longer aliphatic compounds. Hereby, the accumulation of longer aliphatic waxes can induce the formation of larger wax crystals, increasing surface roughness and, thus, hydrophobicity (Heredia-Guerrero et al., 2018). Berry hydrophobicity could be useful as an additional factor for *Botrytis* resilience, as suggested by Deytieux-Belleau et al. (2009), which can be indirectly phenotyped by Z_*REL*_. Conclusively, the fibrous wax structures of ‘Calardis Blanc’ and ‘Norton’ should be investigated regarding increased hydrophobicity. At this point, further high-throughput sensor techniques can be tested for phenotyping grapevine surface characteristics using imaging sensors (e.g., hyperspectral) or structured light (Barré et al., 2019; Haucke et al., 2021).

With a macroscopic look on the glossy surfaces of ‘Morio Muskat’ berries, it is distinctive that the berries barely develop epicuticular waxes. They showed incomplete wax fragments on the berry surface, and it is tempting to speculate that wax synthesis is somehow interrupted during grape berry development. In blueberries (*Vaccinium myrtillus* L.), comparable phenotypes were observed showing changes in the epicuticular wax morphology on the surface of wild types and a glossy type mutant during fruit development (Trivedi et al., 2019, 2020). Trivedi et al. (2019) detected comparable cuticular wax load in both wild type and glossy mutant, but detected variations in the chemical composition and morphology of cuticular waxes along with gene expression for wax biosynthetic genes. So-called glossy mutants are reported, e.g., for tomato fruits (Petit et al., 2014, 2016), leaves of barley *Hordeum vulgare* L. (Luan et al., 2017), rapeseed *Brassica napus* L. (Shirokova et al., 2020), and raspberry *Rubus idaeus* (Pinczinger et al., 2020). Often, glossy plants are described as more susceptible to a variety of insect pests or pathogens. To the best of our knowledge, only very few glossy mutants are known in grapevine yet. One example is ‘Pinot Mouret’ (VIVC15091), which is the glossy mutant of ‘Pinot Noir’ (VIVC9279) showing no wax layer on the berry surface (Maul, 2021). Based on that, the pedigree of ‘Morio Muskat’ was considered. ‘Morio Muskat’ (*Vitis vinifera* ssp. *vinifera* L.) is derived from the crossing of ‘Silvaner’ (VIVC11805) × ‘Muscat A Petit Grains Blanc’ (VIVC8193). Within the genetic repository of Geilweilerhof, the variety ‘Muscat A Petit Grains Blanc’ is planted two rows aside of ‘Morio Muskat’ and shows a comparable phenotype of glossy berry surfaces with only an inconspicuous wax appearance. Thus, we assume that the phenotype of glossy berry surface is inherited from ‘Muscat A Petit Grains Blanc.’ Both varieties are conclusively suitable to study the underlying genetic reasons for glossy berry surfaces in grapevine.

Taken together, the cuticle and its hydrophobic epicuticular wax layer display a promising physical barrier that can reduce the risk for berry cracking and, thus, significantly lower the risk of spreading *Botrytis* bunch rot infection. Berry impedance is a powerful tool to characterize berry surfaces with high-throughput and categorize grapevine varieties, breeding lines, or accessions from a genetic repository regarding resilience to *Botrytis* bunch rot. Such a phenotypic method opens the door to new selection possibilities within breeding programs.

### Quantitative Trait Locus Analysis

Despite the lack of correlations between Z_*REL*_ and berry texture traits (TA_*FORCE*_, TA_*AREA*_, and TA_*GRAD*_), both traits are correlated to *Botrytis* bunch rot infestation and, thus, could be important for the selection of *Botrytis* resilient breeding lines and, finally, molecular marker development. Therefore, both traits were considered for QTL analysis.

In the investigated progeny of ‘Dakapo’ × ‘Cabernet Sauvignon,’ no correlations were detected between berry size and berry texture. Same was observed by Carreño et al. (2015), who also did not find a clear association between berry size and firmness (TA_*AREA*_). As described by Carreño et al. (2015), disassembly of the cell wall within the berry flesh is responsible for changes in berry texture and softening during ripening such as decrease in cellulose and degradation of xyloglucan and pectin. This process is independent from berry size. Concurrently, a correlation between mean berry size and Z_*REL*_ was observed. Genotypes within the investigated population showed both loose bunches with smaller berries and up to very dense bunches with larger berries. Berries within dense bunches often grow closely touching each other; thus, the cuticle is damaged in the contact regions of the berries (Percival et al., 1993). Larger berries and dense grape structure show an increased risk for berry damage due to micro cracks (Becker and Knoche, 2012), favoring *Botrytis* bunch rot infestation (Commenil et al., 1997; Becker and Knoche, 2012). Based on the correlation of berry size and berry impedance, berry size is probably another important key trait affecting berry impedance and, thus, the resilience of grape berries to *Botrytis* bunch rot. Same is observable because of mechanical thinning of grapes in a semi-minimal-pruned-hedge training system resulting in looser grape bunch architecture with smaller berries and reduced infection with *Botrytis* bunch rot (Molitor et al., 2019; Schäfer et al., 2021).

In addition to LG2 and LG11, one major QTL for mean berry diameter (MBD) is located on LG 17. Richter et al. (2019) detected QTLs for mean berry volume in the identical marker VRZAG15 on LG 17 within the population of ‘Calardis Musqué’ × ‘Villard Blanc.’ Doligez et al. (2013) identified QTLs for berry weight on LG 17. Because berry volume, berry weight, and berry size are related to each other, this locus obviously is also involved into the expression of berry size. This locus for berry size was detected in three populations of ‘Calardis Musqué’ × ‘Villard Blanc,’ ‘Dakapo × Cabernet Sauvignon,’ and ‘Riesling × Sauvignon Blanc’ (Rist et al., unpublished). Herzog et al. (2015) published a preliminary QTL on LG17 for Z_*REL*_ with the flanked marker UDV092 that is co-localized to VRZAG15. They did not involve berry size in the QTL analysis. In this study, setting MBD as a co-variant resulted into a shift of the QTL on LG 17 for Z_*REL*_ from VRZAG15 at pos. chr17_6588726 (PN40024.V2) to SCU06 at pos.chr17_3290363 (PN40024.V2). This shift of QTL underlines the relationship between berry size and Z_*REL*_ and the importance of considering mean berry size for phenomics of berry skin traits.

Cuticular waxes are primarily composed of very long chain aliphatic compounds, can contain triterpenoids and metabolites like sterols and flavonoids (Bernard and Joubès, 2013; Dimopoulos et al., 2020), and might influence the expression of Z_*REL*_. With regard to identified QTLs for Z_*REL*_ on LGs 6 and 15, different wax- and cuticle-related genes have been previously identified on these two chromosomes (Mahjoub et al., 2009; Hen-Avivi et al., 2014; Dimopoulos et al., 2020). Mahjoub et al. (2009) described VVMyb5b (*VIT_06s0004g00570*, R2R3-MYB transcription factor) as a regulator of cuticular wax accumulation in tomato that has its key function within triterpenoid biosynthesis. Furthermore, on LG6, CER4-like genes (VIT_06s0080g00110 and VIT_06s0080g00120) were identified, assigned to function as an alcohol-forming fatty acyl-CoA reductase (Dimopoulos et al., 2020). Alcohol-forming fatty acyl-CoA reductases are involved in alcohol forming pathways in which alcohol products can be esterified to fatty acids in order to generate wax esters. Dimopoulos et al. (2020) described three different genes on LG 15, CER1-like, KCS-like, and WSD1-like. Ketoacyl-CoA synthase (KCS)-like *VIT_15s0048g02720* was identified close to the confidence interval on LG15 for Z_*REL*_, TA_*FORCE*_, and TA_*AREA*_. In addition, the QTL on LG15 for TA_*FORCE*_ additional include *VIT_15s0046g00480* and *VIT_15s0046g00490*, both wax ester synthase/acyl-CoA: diacylglycerol acetyl transferase (WSD1). Both were upregulated when VviERF045, a key regulator in berry ripening and putatively involved in cuticular wax biosynthesis, was expressed. Additionally, a strong correlation between the expression of total wax ester and very long chain fatty acid content during berry development was detected (Dimopoulos et al., 2020). To answer the question about the involvement of wax- and cuticle-related genes on the phenotypic expression of berry impedance and berry texture, further fine mapping of detected regions are necessary to detect candidate genes. However, QTL analysis for berry firmness (TA_*AREA*_) is mostly performed on table grape populations where QTLs and candidate genes like *expansin* are identified on LG 8 (Wang et al., 2020), LG 18 (Carreño et al., 2015; Ni et al., 2020; Crespan et al., 2021), and further on LG1, 4, 5, 9, 10, and LG13 (Carreño et al., 2015). Recently, Crespan et al. (2021) identified *VviAGL11* on LG18, one main gene that regulates seedlessness in table grape that seems to be involved in the phenotypic behavior of berry texture. QTLs for berry firmness on LG 2 have not been described yet. As QTLs on LG 2 for Z_*REL*_ and TA_*AREA*_ have disappeared by setting MBD and the Teinturier phenotype as co-variants for MQM mapping, it did not change the QTL on LG 2 for TA_*FORCE*_ and TA_*GRAD*_. Conclusively, this region could be involved in both berry skin firmness and berry skin elasticity. Thus, we assume that berry color locus, i.e., the presence of VvmybA1 and VvmybA2 (Kobayashi et al., 2004; This et al., 2007; Pelsy et al., 2015; Röckel et al., 2020; Sun et al., 2020) could be one additional regulator that is involved in grape berry development. Looking into the confidence interval of 0.3 Mbp on LG2 within the reference genome PN40024 12x.V2 (Table 2) has revealed several MYB-related genes and MYB90 transcription factors and polypeptides. Especially, MYB family members typically function as transcription factors, as MYB proteins might be a key factor in regulatory networks controlling development, metabolism, and responses to biotic and abiotic stresses, as reviewed by Hen-Avivi et al. (2014). They also summarized that in addition to cutin and wax, the cuticle of many fruit species contains secondary metabolites like triterpenoids, sterols, alkaloids, and phenylpropanoids (flavonoids). MYB-type transcription factors regulate the biosynthesis pathways of flavonoid, anthocyanin, and proanthocyanidin in several plant species like maize, apple, and grapevine (Röckel et al., 2020; Sun et al., 2020; Crespan et al., 2021). This is underlined by the observation of Ma et al. (2020) that the MYB family, as one investigated transcription factor, has a potential effect on berry skin firmness. Thus, the berry color locus might enhance berry skin firmness and berry skin elasticity. However, we did not detect the previously published QTL for *Botrytis* bunch rot susceptibility that was mapped on LG 2within a ‘Norton’ (*Vitis aestivalis*) × ‘Cabernet Sauvignon’ population (Sapkota et al., 2019), as we did not map the flanked markers VMC3B10 (PN40024.V2: chr02_5000200) and VMC6F1 (PN40024.V2: chr02_ 8335117). Furthermore, the flanked markers detected in this study (VMC5G7 and GF02-55) are not co-localized to the *Botrytis* bunch rot locus. Looking at the QTL on LG 16 for TA_*GRAD*_ and berry skin elasticity, Guo et al. (2019) described one locus on LG 16 including two calcium-related genes. They describe that calcium is an essential nutrient with an important impact on fruit firmness (Guo et al., 2019). Nevertheless, conducting fine mapping on the investigated progeny, a test on whether selected markers are transferable to plant material with other genetic background and even with white berry color, and gene expression analysis of published wax- and cuticle-related genes needs to be done to support the development of molecular markers for MAS applications of physical traits like Z_*REL*_ and berry texture profile of grapevine berries with resilience to *Botrytis* bunch rot.

## Conclusion

In this study, the physical berry impedance Z_*REL*_ was identified as a reliable indicator for resilience to *Botrytis* bunch rot. Based on the availability of three high-throughput phenotyping methods including berry impedance, berry texture, and grape bunch architecture, plant phenomics for different *Botrytis* bunch rot-related traits is now available, enabling extensive screenings within breeding materials. While the microscopic investigation of cuticle thickness is very labor- and time-intensive, the application of the stated methodologies for cuticle-related traits is fast and objective and, thus, they are promising tools to characterize grapevine varieties in different sites, in genetic repositories, or under different management systems. Until now, *Botrytis* prediction models are still applicable for individual grapevine varieties aiming at improved management using environmental data on temperature, precipitation, air humidity and wetness duration. The combined modeling of Z_*REL*_ and berry texture traits revealed a promising prediction of *Botrytis* bunch rot with *R*^2^*_*McF*_* = 0.99; thus, both traits might be usable in the development of robust *Botrytis* bunch rot forecast systems for screening of breeding materials or new varieties. Beyond Z_*REL*_ and the influence of cuticle thickness, the ultrastructure of epicuticular waxes needs additional research. This includes investigations on variety-specific wax compositions based on different berry impedance values or whether fibrous wax crystals or berry impedance values higher than 1,000 can increase the hydrophobicity of berry surfaces or can affect decreased susceptibility to micro cracks or berry burst. However, grapevine breeding will benefit from both the stated traits and methodologies for seedling selection as well as from the identification of QTLs for each of the traits. Next, fine mapping of detected genomic regions can be conducted to develop molecular markers while high-throughput phenotyping will simplify the confirmation of phenotypic-genotypic expression and will enable a link to the environment because of phenotyping in different sites or years. Because of the observed correlation of the Tenturier phenotype to all the investigated berry traits, mapping populations without that phenotype should be considered. Moreover, in table grapes or other fruit crops like tomato, blueberry, and cherry, *Botrytis* is also an important (post-harvest) disease. Thus, phenotyping Z_*REL*_ and fruit texture probably will also facilitate high-throughput screening and identification of new resilient genotypes of these fruit crops.

## Data Availability Statement

The original contributions presented in the study are included in the article/Supplementary Material, further inquiries can be directed to the corresponding author.

## Author Contributions

KH designed the experimental setup, and managed the scoring and sampling, conducted the Z_*REL*_ measurements, processed and analyzed the phenotypic data for statistics and QTL analysis, conducted the statistical data analysis, and wrote the manuscript. FS conducted the QTL analysis including the calculation of genetic map, improved the BI sensor setup, and edited the manuscript. KH and FS conducted the MQM mapping. H-HK, EB, and MD conducted the Cryo-SEM analysis including sample preparation, microscopic processing, screening, and image capture. OT and RT support the experimental setup with their fundamental knowledge of grapevine breeding, and they provided the plant material at Geilweilerhof. RT, OT, and FS conducted detailed editing of the manuscript. All authors contributed to the article and approved the submitted version.

## Conflict of Interest

The authors declare that the research was conducted in the absence of any commercial or financial relationships that could be construed as a potential conflict of interest.

## Publisher’s Note

All claims expressed in this article are solely those of the authors and do not necessarily represent those of their affiliated organizations, or those of the publisher, the editors and the reviewers. Any product that may be evaluated in this article, or claim that may be made by its manufacturer, is not guaranteed or endorsed by the publisher.

## Footnotes

1
http://www.vivc.de/loci

2
https://integrape.eu/resources/data-management/how-to-describe-a-grapevine-experiment/

## Supplementary Material

Supplementary Figure 1 Ultrastructure of epicuticular wax on the berry surfaces of ‘Riesling’: ‘Calaris Blanc,’ and ‘Norton’ in two independent years of 2018 and 2019 in comparable maturity stage of grapes.

Supplementary Figure 2 Correlation plot of investigated phenotypic traits within the segregating mapping population ‘Dakapo’ × ‘Cabernet Sauvignon’ (D × C) at harvest. The sensor-based phenotypes of mean berry diameter (MBD) as well as physical berry skin characteristics Z_*REL*_. TA_*FORCE*_, TA_*GRAD*_, and TA_*AREA*_ were considered for QTL analysis. MS_*CT*_ and MS_*BST*_ were used as ground truth for berry skin thickness. *N* = 180. MBD, mean berry diameter; Z_*REL*_, relative impedance; TA_*FORCE*_, berry skin firmness; TA_*GRAD*_, berry firmness; TA_*AREA*_, berry skin elasticity; MS_*CT*_, microscopic cuticle thickness; MS_*BST*_, microscopic berry skin thickness.

Supplementary Figure 3 **(A–E)** Boxplot of the berry impedance Z_*REL*_, berry texture traits, and MBD of the mapping population D × C in relation to the expression of the Teinturier phenotype. Different letters indicate significant differences based on Tukey HSD group comparison of means. Green dots display the mean values. *N* = 362. TA, texture analysis; TA_*FORCE*_, berry skin firmness; TA_*AREA*_, whole berry firmness; TA_*GRAD*_, berry skin elasticity; MS_*CT*_, cuticle thickness; MS_*BST*_, berry skin thickness.
