# Supplementary figures and images for: Towards Sensor-Based Phenotyping of Physical Barriers of Grapes to Improve Resilience to Botrytis Bunch Rot
Source: Front Plant Sci. 2022 Feb 10;12:808365. doi: 10.3389/fpls.2021.808365 (PMC8866247; doi:10.3389/fpls.2021.808365)

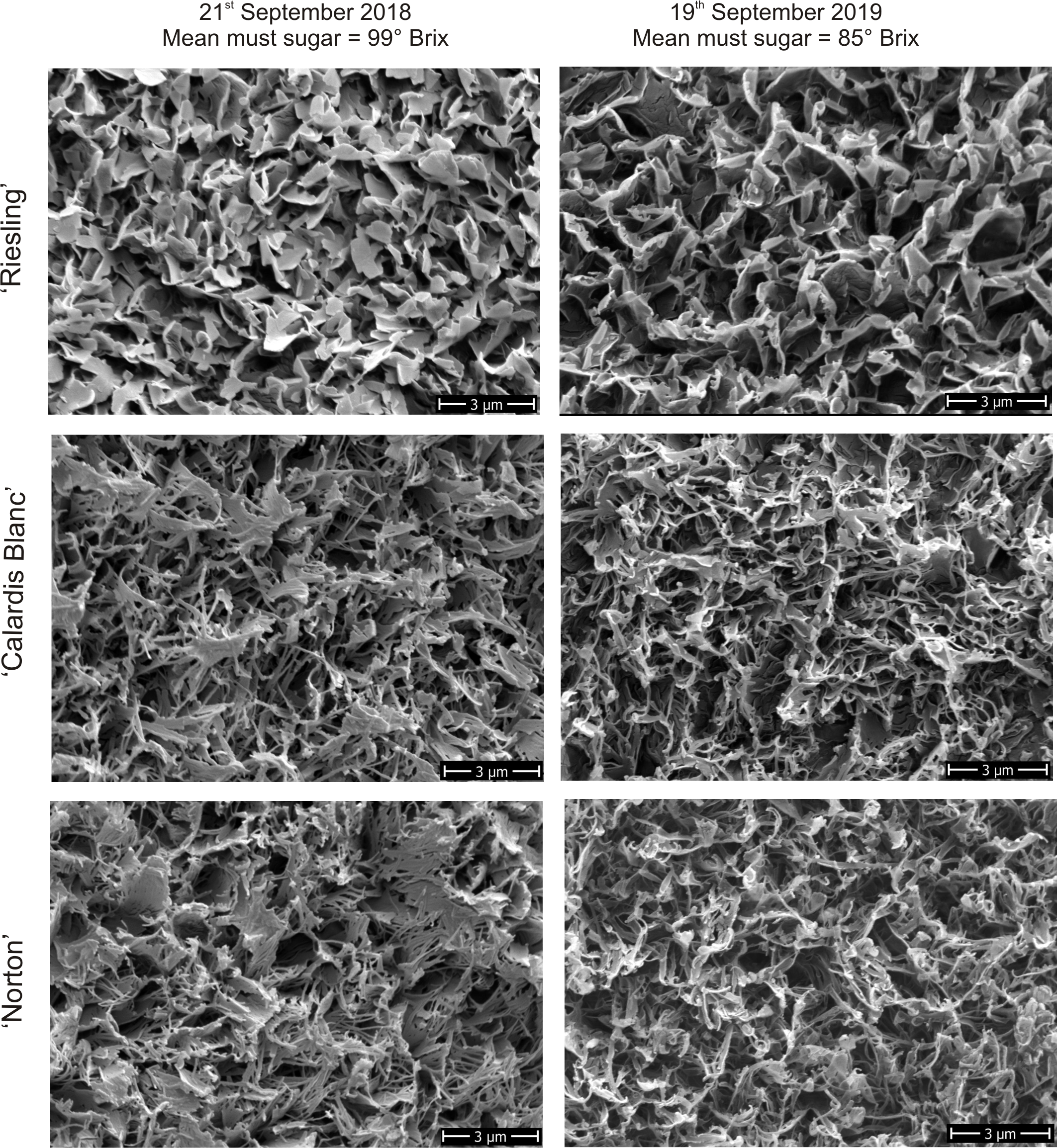

Supplement: Supplementary Figure 1 — Ultrastructure of epicuticular wax on the berry surfaces of ‘Riesling’: ‘Calaris Blanc,’ and ‘Norton’ in two independent years of 2018 and 2019 in comparable maturity stage of grapes. [file Image_1.TIF]

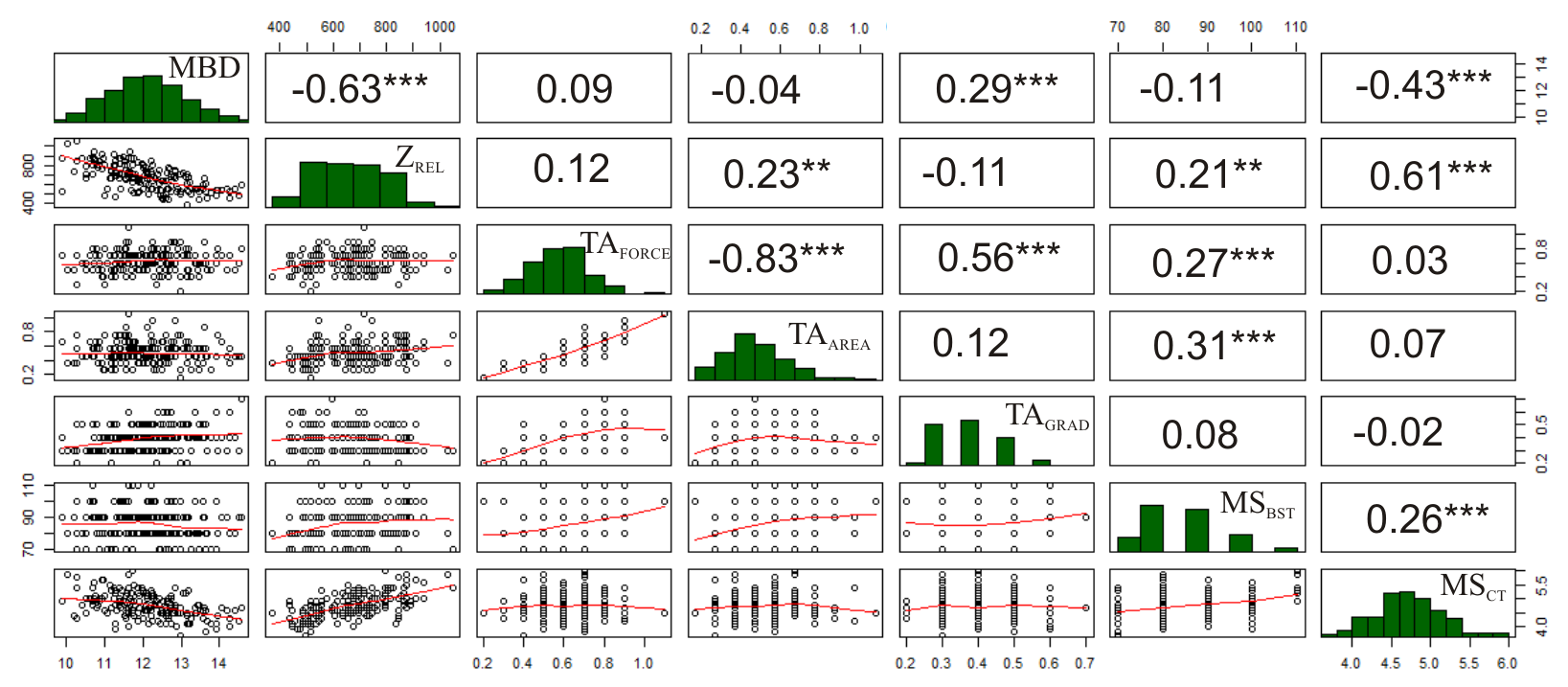

Supplement: Supplementary Figure 2 — Correlation plot of investigated phenotypic traits within the segregating mapping population ‘Dakapo’ × ‘Cabernet Sauvignon’ (D × C) at harvest. The sensor-based phenotypes of mean berry diameter (MBD) as well as physical berry skin characteristics ZREL. TAFORCE, TAGRAD, and TAAREA were considered for QTL analysis. MSCT and MSBST were used as ground truth for berry skin thickness. N = 180. MBD, mean berry diameter; ZREL, relative impedance; TAFORCE, berry skin firmness; TAGRAD, berry firmness; TAAREA, berry skin elasticity; MSCT, microscopic cuticle thickness; MSBST, microscopic berry skin thickness. [file Image_2.TIF]

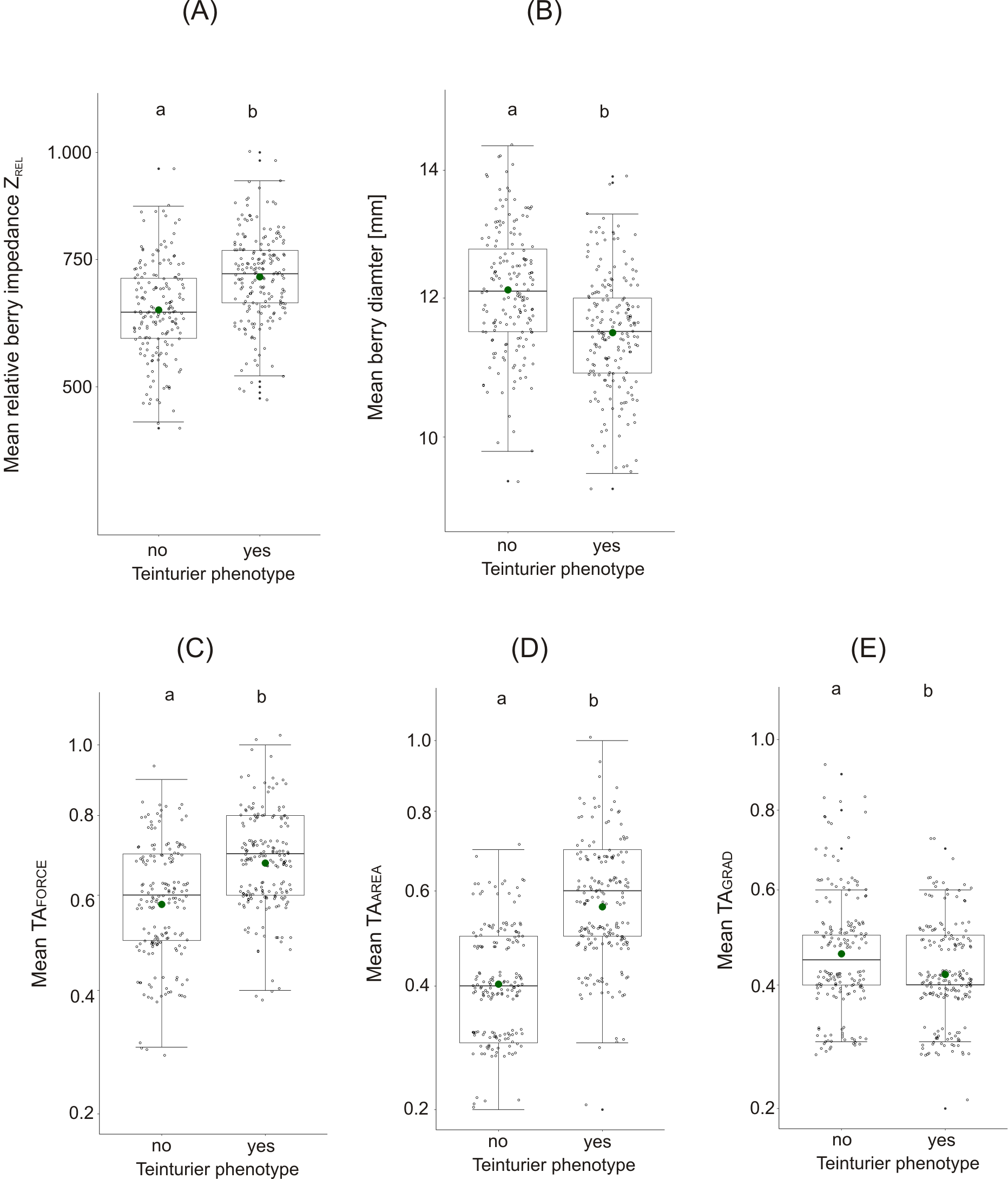

Supplement: Supplementary Figure 3 — (A–E) Boxplot of the berry impedance ZREL, berry texture traits, and MBD of the mapping population D × C in relation to the expression of the Teinturier phenotype. Different letters indicate significant differences based on Tukey HSD group comparison of means. Green dots display the mean values. N = 362. TA, texture analysis; TAFORCE, berry skin firmness; TAAREA, whole berry firmness; TAGRAD, berry skin elasticity; MSCT, cuticle thickness; MSBST, berry skin thickness. [file Image_3.TIF]
